# Prevalence and clinical presentation of molar incisor hypomineralisation among a population of children in the community of Madrid

**DOI:** 10.1186/s12903-024-04003-4

**Published:** 2024-02-13

**Authors:** Sara Ortega-Luengo, Gonzalo Feijóo-Garcia, Mónica Miegimolle-Herrero, Nuria E. Gallardo-López, Antonia M. Caleya-Zambrano

**Affiliations:** 1 Madrid Health Service (SERMAS), Madrid, Spain; 2 Department of Dental Clinical Specialties, Faculty of Dentistry, Complutense University of Madrid, Madrid, 28040 Spain

**Keywords:** Molar incisor hypomineralization (MIH), Enamel developmental defect, Hypomineralised second primary molars (HSPM), Oral Health Units, Pediatric Dentistry

## Abstract

**Objective:**

The main objective of this study was to estimate the prevalence of molar incisor hypomineralisation (MIH), an alteration of tooth enamel with an estimated worldwide prevalence rate of 14%, among children using primary care services in the Community of Madrid, Spain.

**Materials and methods:**

This was a descriptive, cross-sectional and multicentre study. After calibrating all researchers and following the diagnostic criteria of the European Academy of Paediatric Dentistry (EAPD), children aged between 8 and 16 years who were users of the dental services at 8 primary oral health units of the Madrid Health Service (SERMAS) were included. The children underwent a dental examination, and the parents were asked to complete a questionnaire.

**Results:**

The prevalence of MIH was 28.63% (CI: 24.61–32.65%). The age cohorts most affected by MIH were 8 years (21.4%) and 11 years (20.7%). The presence of MIH was greater among girls (85; 60.71%) than among boys (55; 39.28%). The mean number of affected teeth per patient was 4.46 ± 2.8. The most frequently affected molar was the upper right first molar (74.3%), and the upper left central incisor was the most affected incisor (37.85%). Opacities were the defects most frequently recorded (63.57%).

**Conclusions:**

The prevalence of MIH in this study is the highest of all relevant studies conducted in Spain.

## Introduction

Dental enamel alterations have been studied since 1895 [1]. One of these alterations is molar incisor hypomineralisation, described for the first time at the end of the 1970s in Sweden, when paediatric dentists began to detect changes in the enamel of first molars and permanent incisors, is an alteration of the tooth enamel of unknown aetiology and not related to dental caries [2]. In 2001, Weerjheim et al. suggested using the name molar incisor hypomineralisation (MIH), which was defined as “hypomineralisation of systemic origin of one to four permanent first molars frequently associated with affected incisors” and must be differentiated from other enamel pathologies, such as diffuse opacities, hypoplasia, amelogenesis imperfecta or non-MIH hypomineralisations, such as initial caries [3–5].

In 2003, the European Academy of Paediatric Dentistry (EAPD) established the diagnostic criteria for MIH: demarcated opacity, posteruptive enamel breakdown, atypical restauration and extracted molar due to MIH, and the first prevalence studies were carried out in Europe to establish strategies for the diagnosis, treatment and prevention of this pathology [6, 7].

Microscopically, compared with healthy enamel, the enamel of affected teeth shows porous areas with a higher carbon content and lower calcium and phosphorus concentrations. The borders between healthy and affected enamel are well defined. At the macroscopic level, affected teeth present alterations in enamel translucency, resulting in marked opacities, in addition to areas with fractured enamel and exposed dentin [8]. Clinically, patients affected by MIH present with several problems. Frequently, mild cases only show aesthetic defects, especially when anterior teeth are affected. More serious cases involve tooth sensitivity to changes in temperature and during brushing, resulting in inadequate oral hygiene, with a consequent increase in number and severity of caries and gingival problems. Dental professionals will observe rapid tooth decay, especially in posterior sectors, with lesions in unusual areas, making restorative treatment difficult and potentially causing premature tooth loss. In addition to the above, anaesthetic management of patients may be challenging due to chronic pulp inflammation resulting from continuous bacterial penetration from the porous enamel into the dentin, which can cause behavioural problems during dental treatment [6, 9, 10]. Aetiology of MIH is unknow and has been related with environmental factors presents from the last months of pregnancy to the first years of life that are capable of altering the enamel development, such as, maternal illness, early childhood diseases or use of drugs, included self-medication [11, 12]. 

Research on the prevalence of MIH began in 2000, but despite the popularisation of the use of the EAPD criteria, the results were substantially different among investigations; as such, several researchers highlighted the need to standardize the criteria among studies [13, 14]. In 2015, Ghanim et al. proposed a new evaluation system that takes into account both the clinical status of enamel lesions and their extent [15]. Currently, the prevalence of MIH is considered high. 2 recent reviews estimated worldwide prevalence rates of 13.1% and 14.2% [16, 17]. In Spain, the estimated prevalence rate is 20% [17, 18]. Regarding the Community of Madrid, the estimated prevalence is approximately 12% [19], but there are no published studies that follow the standardized assessment criteria recommended by the EAPD for MIH studies.

In our setting, public dental care in the Community of Madrid, which is provided through the Oral Health Units (OHUs) of Primary Care Health Centres, there are also no studies on the prevalence of MIH that meet the EAPD criteria. Considering that this pathology has a high prevalence and is a complex clinical problem and given that dental consultations in the Madrid public health system mainly involve preventive care, this study was designed with the aim of determining the prevalence of MIH among children users of public dental services in Madrid.

## Materials and methods

This was an observational, descriptive, cross-sectional and multicentre study to estimate the prevalence of MIH among children aged between 8 and 16 years in different oral health units (OHUs) of the Community of Madrid, Spain. We selected this age group because it is the group included in public oral health program. This study was approved by the Ethics Committee of the Hospital Clínico San Carlos following the Declaration of Helsinki’s ethical principles for medical research involving human subjects (Internal Code: 21/162-E_Tesis).

### Study population and sample selection

Participation was voluntary; the guardians of the children were approached about participating in the study, and all those who agreed signed an informed consent form. The inclusion criteria were as follows: (1) children between 8 and 16 years of age, (2) complete eruption or eruption of at least 1/3 of the occlusal surface of the first 4 permanent molars, and (3) signed informed consent form (guardians).

The exclusion criteria were as follows: (1) no first permanent molars or eruption less than 1/3 of the occlusal surface of molars, (2) fixed orthodontic appliances, (3) systemic diseases or under treatment with drugs that could alter enamel development, and (4) lack of cooperation during the examination.

The sample size was calculated for an estimated prevalence of 12%, referencing a previous study carried out in a population similar to ours [14], with a precision of 3% and a confidence level of 95%, which resulted in a sample size of 451 children.

The OHUs of the Madrid Health Service (SERMAS) were divided into two groups to facilitate randomization:

- 40 metropolitan OHUs located in the city of Madrid.
- 46 OHUs located in other municipalities of the Community of Madrid. Those located in towns with fewer than 20,000 inhabitants were considered rural OHUs.

Ultimately, 8 OHUs were selected: 4 in the city of Madrid and another 4 in the municipalities of Parla, Alcobendas and Algete (rural).

### Training and calibration

**Fig. 1:**
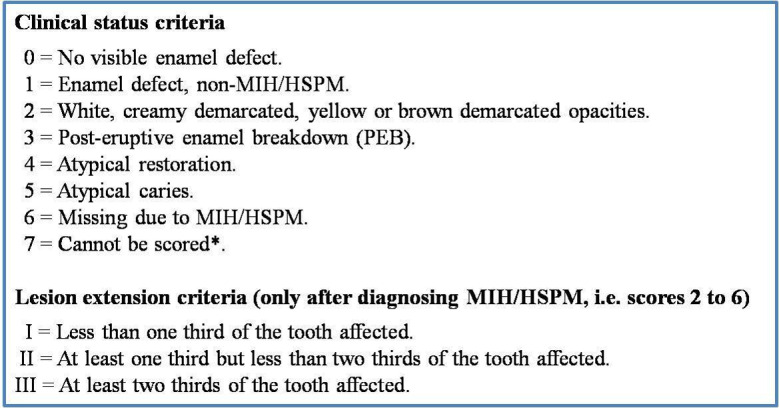
EAPD diagnostic criteria for MIH

Prior to data collection, the 8 participating dentists received a training session on the diagnosis of MIH using the EAPD criteria [10] (Fig. 1). Subsequently, calibration was carried out. A questionnaire with 29 photographs of teeth was sent to each dentist; the dentists provided diagnoses using the EAPD diagnostic criteria on which they were previously trained. Each dentist completed the questionnaire twice, within a week between each session. The kappa coefficient was used to calculate inter-examiner agreement. All kappa values were between 0.74 and 0.94; therefore, according to the Landis and Koch scale, the degree of agreement was classified as good and very good.

### Data collection

Data collection consisted of 2 steps: a dental examination and a questionnaire to be completed by the parents. The oral examination was performed by the dentists in an office dental chair. The light source was the equipment light. Cotton balls were used to dry oral surfaces; an air syringe was not used. Ball-ended probes were used to detect irregularities in the enamel surface. Each dentist completed a data collection sheet for each patient.

### Data analysis

IBM SPSS Statistics version 27.0 was used for statistical analyses. Quantitative variables are described using means and standard deviations, and categorical variables are described as frequencies and percentages. The prevalence of MIH was calculated as a percentage, and estimations are reported using confidence intervals. The Mann‒Whitney U test and the Wilcoxon test were used to analyse significant differences between different samples, and the chi-square test was used to determine the differences between percentages. Differences were considered statistically significant when *p* < 0.05.

## Results

The sample comprised 489 children between 8 and 16 years of age, of whom 258 (52.76%) were girls and 231 were boys (47.23%). A total of 140 children were affected by MIH; therefore, the estimated prevalence of MIH in this study was 28.63% (CI: 24.61–32.65%). Regarding sex, 55 boys (39.28%) and 85 girls (60.71%) were affected by MIH. Girls were affected more than boys and difference was significant (chi-square test, *p* < 0.05).

**Fig. 2:**
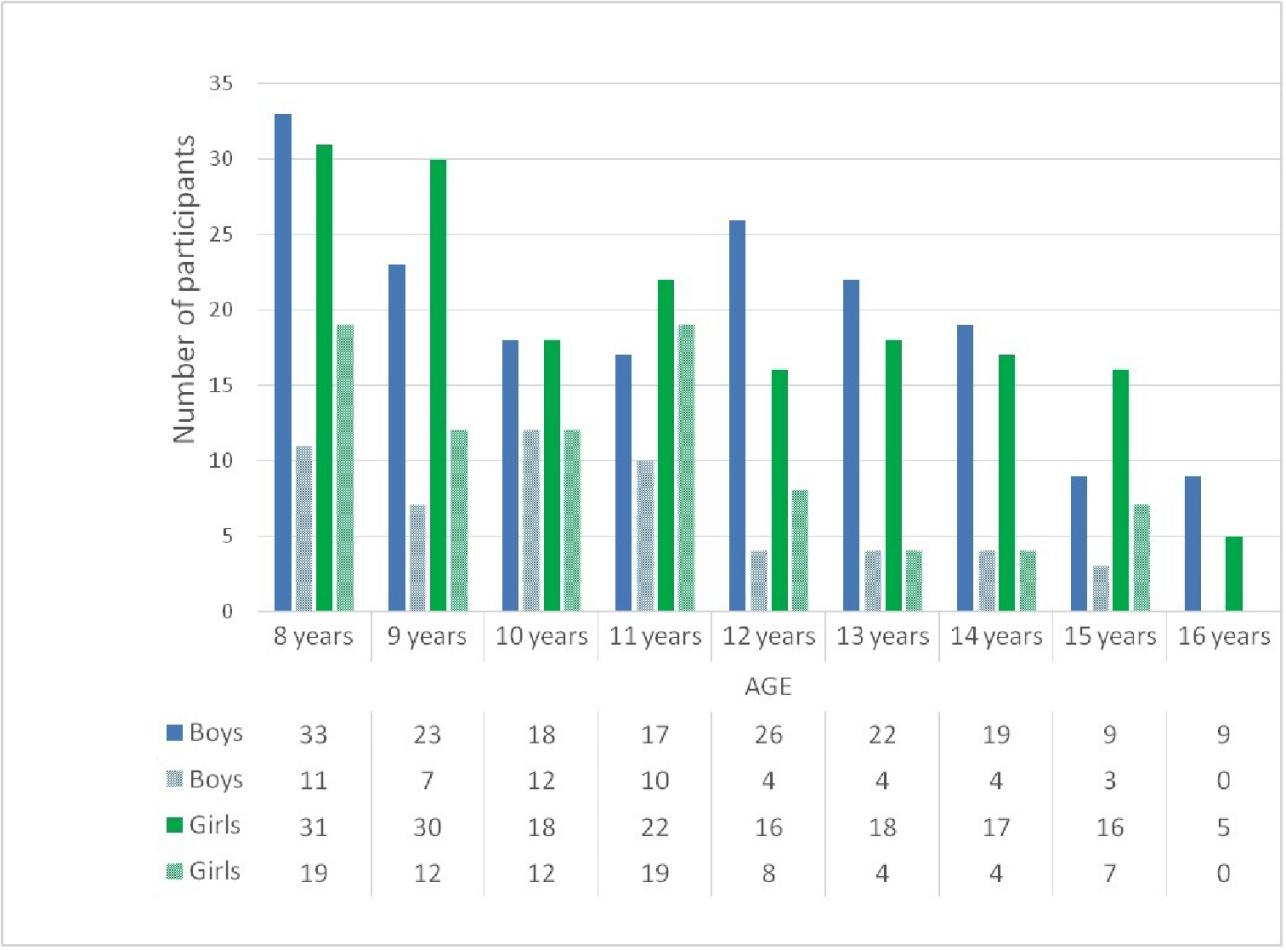
Distribution of MIH in the sample. Age and Sex

The mean age of the children (*N* = 489) at the time of the examination was 11.01 ± 2.3 years, and that of children affected by MIH (*N* = 140) was 10.5 ± 2.11 years. The age cohorts most affected by MIH were 8 years (21.4%), 11 years (20.7%) and 10 years (17.1%); notably, in the 16-year-old cohort, no patients presented MIH. There were significant differences between the 10-year-old cohort and 11-year-old cohort and other cohorts with regard to the presence of MIH (chi-square test, *p* < 0.05). Figure 2 shows the distribution of the sample and the prevalence of MIH by sex and age at the time of the examination.

A total of 1,672 permanent index teeth were studied. The number of affected teeth in each patient with MIH varied between 1 and 12; the mean number of affected teeth was 4.46 ± 2.8. In the upper arch, 55.6% (*n* = 347) of the teeth presented hypomineralisation, and the average number of affected teeth was 2.47 ± 1.66. In the lower arch, the percentage of affected teeth was lower, 44.39% (*n* = 277), with a mean of 1.99 ± 1.55 affected teeth. In both arches, the most common number of affected teeth was 2, and 6 patients had 12 affected index teeth. Significant differences were found between the upper and lower arches with regard to the mean number of affected teeth (Wilcoxon signed rank test, *p* = 0.001).

The most frequently affected molar was the upper right first molar (74.3%, *n* = 104), followed by the upper left first molar (68.6%, *n* = 96), the lower left first molar (65%, *n* = 91) and the lower right first molar (61.4%, *n* = 86); the least frequently affected tooth was the upper left lateral incisor (14.3%, *n* = 20).

Regarding the number of affected molars in each patient with MIH, the mean number of affected molars was 2.69 ± 1.15. A total of 36.4% (*n* = 52) showed involvement of the 4 molars, 30% (*n* = 42) had 2 affected molars, 18.6% (*n* = 26) had a single affected molar, and 14.3% (*n* = 20) had 3 affected molars. The mean number of affected incisors in patients with MIH was 1.76 ± 2.1. 36,4% of these patients (*n* = 51) showed no affected incisors and had hypomineralisation only on the first permanent molars or molar hypomineralisation (MH). On average, 1.03 ± 1.31 incisors were affected in the upper arch, and 0.75 ± 1.13 were affected in the lower arch. Significant differences were found in the number of affected upper and lower incisors (Wilcoxon signed rank test, *p* = 0.004). The distribution of incisor involvement by the number of affected molars is shown in Table 1.

**Table 1:** Distribution of MH and MIH affected incisor and molars

Number affected molars	Number affected incisors									TOTAL (%)
	0	1	2	3	4	5	6	7	8	
**1**	10	6	6	1	1	0	0	0	0	**24** (17.8)
**2**	24	6	6	4	0	0	1	0	0	**41** (30.4)
**3**	7	7	3	3	0	0	0	0	0	**20** (14.8)
**4**	10	6	8	6	9	1	2	2	6	**50** (37)
**TOTAL** **(%)**	**51** (37.8)	**25** (18.5)	**23** (17)	**14** (10.4)	**10** (7.4)	**1** (0.7)	**3** (2.2)	**2** (1.5)	**6** (4.4)	**135** ^**a**^ (100)

^a^Not erupted index teeth are not included

Regarding types of defects due to MIH, those proposed by the EAPD— demarcated opacities, post-eruptive enamel breakdown, atypical restorations, atypical caries and missing teeth—were analysed. Of the 1,672 teeth analysed, 624 (37.32%) showed some type of enamel defect. A total of 519 (31%) presented opacities, 47 (2.8%) presented atypical restorations, 33 (2%) presented post-eruptive enamel breakdown, 24 (1.4%) presented atypical caries, and 1 tooth (0.1%) had been extracted because of MIH.

In patients with MIH (*n* = 140), opacity was the most frequent defect (*n* = 76) in the upper first molars. Tooth loss associated with MIH was detected only in 1 lower left first molar.

Regarding primary molars, 97 patients (69.3%, *n* = 140) had at least 1 s primary molar present at the time of the examination, and 74 (52.9%) had all 4. Of those who had a primary molar (*n* = 97), 73 (75.3%) did not present any alteration in the molars present, 9 (9.3%) had 1 molar affected by hypomineralised lesions on second primary molars (HSPM), 9 (9.3%) had 2 affected molars, 2 (2.1%) had 3 affected molars, and 4 (4.1%) had 4 affected molars. Regarding the type of defect, 341 primary second molars were analysed. A total of 39 teeth (11.4%) had opacities, 6 (1.8%) had atypical caries, 3 (0.9%) had atypical restorations, and 1 (0.3%) had post-eruptive enamel breakdown. Table 2 shows the percentages of affected index teeth by defect type.

**Table 2:** Distribution of defect types in permanent index teeth

INDEX TEETH	Not affected (%)	Affected (%)						TOTAL
		Demarcated opacity	Post-eruptive enamel breakdown	Atypical filling	Atypical caries	Extracted due to MIH	Not erupted	
**16**	36 (25,7)	76 (54.3)	7 (5)	13 (9.3)	8 (5.7)	0 (0)	0 (0)	**140 (100)**
**12**	117 (83.6)	22 (15,7)	0 (0)	0 (0)	0 (0)	0 (0)	1 (0.7)	**140 (100)**
**11**	87 (62.1)	51 (36.4)	2 (1.4)	0 (0)	0 (0)	0 (0)	0 (0)	**140 (100)**
**21**	88 (62.9)	50 (35.7)	2 (1.4)	0 (0)	0 (0)	0 (0)	0 (0)	**140 (100)**
**22**	117 (83.6)	20 (14.3)	0 (0)	0 (0)	0 (0)	0 (0)	3 (2.1)	**140 (100)**
**26**	44 (31.4)	74 (52.9)	6 (4.3)	10 (7.1)	6 (4.3)	0 (0)	0 (0)	**140 (100)**
**36**	49 (35)	67 (47.9)	6 (4.3)	12 (8.6)	5 (3.6)	1 (0.7)	0 (0)	**140 (100)**
**32**	116 (82.9)	23 (16.4)	0 (0)	0 (0)	0 (0)	0 (0)	1 (0.7)	**140 (100)**
**31**	115 (82.1)	25 (17.9)	0 (0)	0 (0)	0 (0)	0 (0)	0 (0)	**140 (100)**
**41**	117 (83.6)	23 (16.4)	0 (0)	0 (0)	0 (0)	0 (0)	0 (0)	**140 (100)**
**42**	109 (77.9)	28 (20)	1 (0.7)	0 (0)	0 (0)	0 (0)	2 (1.4)	**140 (100)**
**46**	53 (37.9)	60 (42.9)	9 (6.4)	12 (8.6)	5 (3.6)	0 (0)	1 (0.7)	**140 (100)**

The extent of the defects and their severity were evaluated using the EAPD criteria: Type I was considered when defect extension was less than 1/3 of the affected tooth, type II when at least 1/3 but less than 2/3 of the affected tooth and type III when 2/3 or more of the tooth was affected. Of the teeth analysed in which the size of the defect was recorded (*n* = 613), 51.71% of the defects showed type I extension, 35.7% showed type II extension, and 12.56% showed type III extension.

With regard to the severity of the affected teeth (*n* = 624), 83.17% (*n* = 519) of teeth only showed changes in colour, i.e., mild severity, and 16.82% (*n* = 105) of the teeth presented severe disease, i.e., post-eruptive enamel breakdown, atypical caries or restorations or loss due to MIH. Of the 140 patients diagnosed with MIH, 61 (43.57%) had severe disease in at least 1 tooth, and in 79 patients (56.42%), all the teeth presented mild disease. In the children with mild disease, an average of 3.84 teeth were affected, and in the children with severe disease, an average of 5.28 teeth were affected. The difference in the mean number of affected teeth as a function of severity was significant (Mann‒Whitney U test for independent samples, *p* = 0.003). The most severe lesions were found in older children (mean age of 11.15 years for severe lesions versus 10.25 years for mild lesions (Mann‒Whitney U test for independent samples, *p* = 0.014). No relationship was found between lesion severity and sex.

**Fig. 3:**
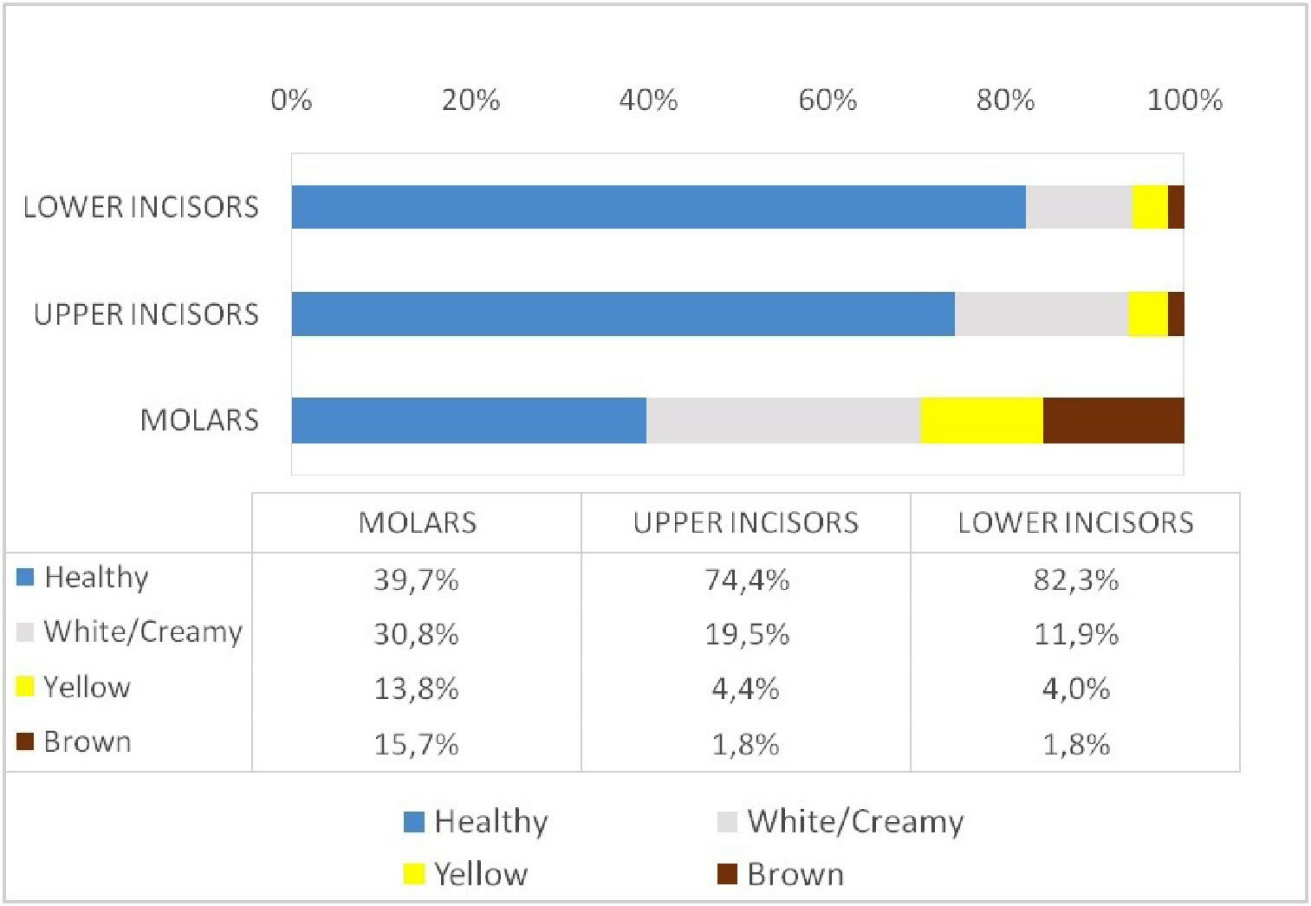
Healthy and demarcated opacity teeth. Color of the opacities according to the type of index tooth (%)

Regarding defect colour, most of the teeth with opacities were white. Colour alterations were analysed in 515 teeth. A total of 60.97% (*n* = 314) of teeth had white defects, 21.16% (*n* = 109) had yellow defects, and 17.86% (*n* = 92) had brown defects. Yellow and brown defects occurred more frequently in molars than in incisors. Figure 3 shows the percentage of opacities by defect colour and tooth type.

## Discussion

The main objective of this study was to estimate the prevalence of MIH among users of public dental services in Madrid, Spain. Worldwide, the prevalence of MIH is highly variable, ranging from 0.5 to 40% () [17, 20, 21]. In Spain, it ranges from 12 to 24.8% [17–19, 22, 23], higher than the prevalence in other areas of Europe such as Croatia (13%) [24], Germany (13.5%) [25], Sarajevo (11.5%) [26], Switzerland (14.8%) [27]and Belgium (18.6%) [28]. Although EAPD was the first to unify diagnostic criteria for MIH, others are currently accepted [29]. In our case, we decided to use EAPD criteria because we were more interested in knowing prevalence of MIH than in treatment needs.

In this study, the first to be carried out in Madrid using the EAPD criteria, the estimated prevalence of MIH among children aged between 8 and 16 years was 28.63%. This result is higher than expected based on studies carried out in our country; however, the results were similar to those reported in other studies in Caracas (Venezuela) (25.35%) [30], Brazil (28.7%) [31], Mexico (35.4%) [32] and Lebanon (26.7%) [33]. Similar to our study, others have also investigated the presence of MIH among public health care users. Rodriguez-Rodriguez et al. (Venezuela) and López Jordi et al. (Argentina and Uruguay) studied the prevalence of MIH among public and private health care users and found higher prevalence rates in the private system. These authors explain these higher rates as follows: in their countries, only people from the lowest socioeconomic level and who do not receive social security benefits seek dental care through the public system [30, 34]. In contrast, in our study, conducted only with participants who use the public system, we obtained higher prevalence rates than those obtained in any other study conducted in our country. We believe that this is because more children with dental pathologies are seen through the public system in Madrid because the Madrid health system, which provides universal and fully subsidized medical coverage, provides free dental treatment for permanent teeth in children between 7 and 16 years of age [35].

With regard to sex, although several recent meta-analyses have not found differences [16, 17, 20], in our study, we found a higher prevalence of MIH among girls, a finding that is similar to the results reported in other published studies [24, 26, 36, 37]. Schwendicke et al., in a meta-analysis carried out in 2018, did not find differences between sexes; however, they acknowledge that in certain areas, the prevalence among girls is significantly higher than among boys, but the causes could not be determined [16]. Chawla et al. [38] suggest that this higher prevalence may be observed because dental maturity usually occurs earlier in girls, and therefore, their teeth would have been erupted for longer, with more post-eruptive enamel breakdown detected. Being MIH an alteration in which there may be an aesthetic affectation, especially when the incisor group is affected, we believe that the greater presence of MIH n girls in our study can be explained because girls tend to show more concern about their physical appearance. All participants in this research were users of a free access preventive program and, affected girls, would attend dental check-ups more frequently than those who are not affected.

In studies in which different age groups were analysed, the prevalence of MIH seems to be influenced by the age of the participants. According to other studies, the prevalence of MIH appears to be higher in younger patient cohorts. This may be because at 8 years of age, the age recommended by the EAPD for conducting MIH prevalence studies [21], the first permanent molars erupt, and most are still intact without enamel breakdown; this allows the visualization of opacities, facilitating the diagnosis of MIH. In older children, it is possible that more post-eruptive enamel breakdown occurs, and if conservative treatment is administered, these molars may not be identified as affected by MIH, resulting in the underdiagnosis of this condition [10, 21]. In our study, the highest prevalence of MIH corresponded to the youngest children, 8 years of age (21.4%), similar to the studies by Grieshaber et al. [27] and Arslanagic-Muratbegovic et al. [26]; however, in this study, the age of participants in the youngest cohort was 6 years. Our study found significant differences between the 10-year-old and 11-year-old cohorts and the other cohorts; however, this may have been influenced by the sample size because some studies propose that each cohort should be made up of at least 100 children [21]; in this study, the size of the cohorts was less than 100.

On the other hand, youngest children who still have primary molars may present defects in the enamel of these teeth, comparable to MIH, which is called hypomineralisation of second primary molars (HSPM). It has been shown to be a pathology predictive of MIH [5].

Regarding the possibility of greater MIH involvement in the upper or lower arch, studies are not conclusive. Several investigations have shown greater involvement in the upper arch than in the lower arch [26, 31, 39–41] but found no significant differences; in this study, the number of teeth affected by MIH was also greater in the upper arch than in the lower arch, but the difference was significant. Chawla et al. (2008) provided various reasons for greater maxillary molar involvement. The lower molars tend to erupt earlier than the upper molars; therefore, as the former have been exposed to the oral environment for longer, they can be affected earlier by enamel breakdown, with such patients receiving conservative treatment, leading to underestimations of the diagnosis of MIH in the lower molars. Additionally, examinations performed in the lower arch may be influenced by the presence of the tongue, which can make it difficult to detect MIH lesions in the lower molars [38].

The results of this study agree with those reported in many other studies, i.e., opacities are the most frequent lesions [25, 26, 41, 42]. In our study, atypical restorations ranked second, which differs from other studies, which found post-eruptive enamel breakdown more frequently) [26, 39, 43]. Given that the children who participated in our study were users of an oral health programme that includes restorative treatment of permanent molars, we believe that the reason for the presence of atypical restorations as the second most frequent lesion is that teeth with post-eruptive enamel breakdown received restorative treatment. Similar to García-Margarit et al. [22], we believe that longitudinal designs are needed to assess the progression of lesions over time.

To analyse lesion extension, the scale proposed by Ghanim et al. [15] was used, and less than 1/3 of the tooth surface affected was the most frequently observed extension, similar to results reported in the studies by Argote et al. [41] and Sidhu et al. [44]. The lesion severity most frequently found in our study was mild, with results comparable to those obtained in other studies [30, 42–44].

Patients with more severe lesions had a greater number of affected teeth and were older than patients with mild lesions, findings that are consistent with the results of other studies [26, 41]. In this study, examinations were performed in an office dental chair, which is why we believe that the results may be more reliable than those reported in studies in which examinations were performed in schools and without the chair light. The limitations of our study include the use of the short data form suggested by the EAPD, which is why only the index teeth were analysed, and the fewer number of participants in the oldest age groups, which decreases the representativeness of the sample.

## Conclusions

The prevalence of MIH among children using public dental services in Madrid was 28.63%, the highest among the studies that have been carried out in Spain. The prevalence of MIH was highest among female patients between the ages of 8 and 11 years. Opacities were the most frequent lesions, followed by atypical restorations. Regarding the extent of the defects, most of the teeth showed less than 1/3 of the tooth surface affected. Brown defects were more prevalent in molars, and the incisors showed white or yellow defects. Longitudinal studies are needed to measure lesion progression. Given the high prevalence of MIH, it is necessary to promote multidisciplinary awareness and prevention programs for primary health care personnel, including nurses and paediatricians.

## Data Availability

All collected data from patients analysed during this study are included in this published article.

## Abbreviations

EAPD: European Academy of Paediatric Dentistry
MIH: Molar Incisor Hypomineralisation
OHUs: Oral health units
